# Prevalence of reported incidental adrenal findings in chest computerized tomography scans performed during the COVID-19 pandemic in a single center in Northeast Brazil

**DOI:** 10.20945/2359-3997000000592

**Published:** 2023-01-25

**Authors:** Lucas José Tavares de Magalhães, Victor Gomes Rocha, Thiago Costa de Almeida, Edoarda Vasco de Albuquerque Albuquerque

**Affiliations:** 1 Centro Universitário Tiradentes Maceió AL Brasil Centro Universitário Tiradentes (UNIT), Maceió, AL, Brasil.; 2 Hospital Memorial Arthur Ramos Maceió AL Brasil Hospital Memorial Arthur Ramos, Maceió, AL, Brasil.

**Keywords:** Adrenal incidentaloma, CT scan, adrenal gland

## Abstract

**Objective:**

We investigated the prevalence of adrenal incidentalomas (AIs) in a nonselected Brazilian population in chest computed tomography (CT) performed during the COVID-19 pandemic.

**Materials and methods:**

This was a retrospective cross-sectional observational study using chest CT reports from a tertiary in- and outpatient radiology clinic from March to September 2020. AIs were defined by changes in the shape, size, or density of the gland initially identified in the released report. Individuals with multiple studies were included, and duplicates were removed. Exams with positive findings were reviewed by a single radiologist.

**Results:**

A total of 10,329 chest CTs were reviewed, and after duplicate removal, 8,207 exams were included. The median age was 45 years [IQR 35-59 years], and 4,667 (56.8%) were female. Thirty-eight lesions were identified in 36 patients (prevalence 0.44%). A higher prevalence was observed with age, with 94.4% of the findings in patients aged 40 years and over (RR 9.98 IC 2.39-41.58, p 0.002), but there was no significant difference between the sexes. Seventeen lesions (44.7%) had more than 10 HU, and five lesions (12.1%) were more than 4 cm.

**Conclusions:**

The prevalence of AIs in an unselected and unreviewed population in a Brazilian clinic is low. The impact on the health system caused by AIs discovered during the pandemic should be small regarding the need for specialized follow-up.

## INTRODUCTION

Adrenal incidentalomas (AIs) are clinically silent lesions discovered by imaging studies performed for reasons other than suspected adrenal disease ( 1 – 7 ). Such findings can represent a nonfunctioning adenoma, a lesion without major clinical repercussions, to a hormone-producing adenoma or carcinoma, the latter with high morbimortality, or even lesions not directly related to the adrenal glands, such as metastasis ( 2 – 4 ). Clinically relevant incidentalomas necessitate specialized consultation and follow-up, as well as hormonal investigation ( 4 ).

The prevalence of AI varies depending on patient selection and the primary data source and is higher in autopsy than in radiological series ( 3 ). Due to the low- resolution technology available in the 1980s, the overall detection of AI in the first published CT scan series was approximately 0.6%, increasing to a prevalence of approximately 4%-5% as the CT resolution improved ( 2 , 8 – 10 ). On the other hand, most recent AI studies included older patients and had structured protocols in which the CT scans were later thoroughly reviewed by specialized radiologists, which could represent a selection bias ( 11 ). In the early 2000s, a prevalence of 2.5% was reported in Brazil, which was significantly higher in males and increased with age ( 1 ).

During the COVID-19 pandemic in Brazil, despite the recommendations not to use imaging exams routinely for viral infection screening ( 12 – 14 ), chest CT scans were widely used in the emergency room for clinical decision- making. Considering the prevalence of AI in previous reports, the massive number of chest CT scans performed could generate a great burden on the health system due to the need for specialized follow-up and hormonal screening for clinically relevant lesions ( 4 ). Additionally, individuals below 30 years of age usually do not have medical issues requiring high-resolution imaging scans of the chest or abdomen. Since COVID-19 infection does not have an age bias, this younger population, which is more prone to disobey social distancing measures, would have high infection rates with a mild presentation. Despite not being indicated ( 12 – 14 ), this population still often underwent chest CT scans to determine the need for hospital admission.

In this setting, the aim of this study was to investigate the prevalence of AI in a nonselected population in Brazil, utilizing chest CT scans performed due to suspected COVID-19 infection in a private radiology clinic that attends outpatients and tertiary care inpatients, including younger individuals who routinely are not submitted to imaging assessment.

## MATERIALS AND METHODS

### Participants and sample size

All chest CT scans performed in subjects aged 18 years or above in a private radiology center from March to September 2020 were included. The radiology center is located on the grounds of one of the main private tertiary care hospitals in Maceió, Brazil but also attends outpatients from private clinics throughout the city. The mean number of CTs performed was approximately 1,386 exams per month, escalating to a mean of 2,382 exams per month in 2020. Inpatients, including infirmary, ICU, and emergency rooms, represent 50% of the usual demand, and the other half is composed of outpatients from the hospital itself and other private clinics in the city. The current rate of report rectifications is 0.2%.

This study was approved by the local ethics committee (CAAE 48312821.6.0000.5641). The sample size was calculated as 1,623 CT scans to obtain a 2.5% prevalence of adrenal incidentaloma with a 99% confidence interval and an estimated error of 1% (15).

### Incidentaloma identification

A chest CT scan was chosen due to its wide use during the COVID-19 pandemic and its capability to visualize the adrenal glands. Moreover, a chest CT scan in this setting would prevent the selection of patients whose purpose of the radiological study could be related to the adrenal gland, i.e., an identified lesion would not be a true incidentaloma. Individuals who underwent multiple chest CT scans had only their first exam included in the analysis.

All helical chest CT scans were performed in the 16- Slice GE Optima CT520 CT Scanner (GE Healthcare) and were unenhanced at a 5 mm slice thickness and 1.25 mm reconstruction interval. AI was defined by a change in the shape, size or density of one or both adrenal glands, first identified in the released report. All scans with an incidental adrenal finding were then revised by a single radiologist with 10 years of experience in internal medicine scan analysis, and data regarding the size and tomographic density in Hounsfield Units (HU) were collected, as well as information about the gender and age of all participants. AIs are considered benign when they are lipid-rich, i.e., have less than 10 HU in a noncontrast CT scan and are less than 4 cm (4).

### Statistical analysis

Continuous variables were described as the mean with standard deviations or the median with interquartile ranges. Categorical variables such as sex and age (<40 years, 40-60 years, >60 years) were compared using the chi-square test. Logistic regression was used to assess variables with a higher prevalence of AI. A P value < 0.05 was considered statistically significant. All statistical analyses were performed using SPSS Statistical Package, version 21.0 (IBM Corp., Armonk, NY, USA).

## RESULTS

**Figure 1:**
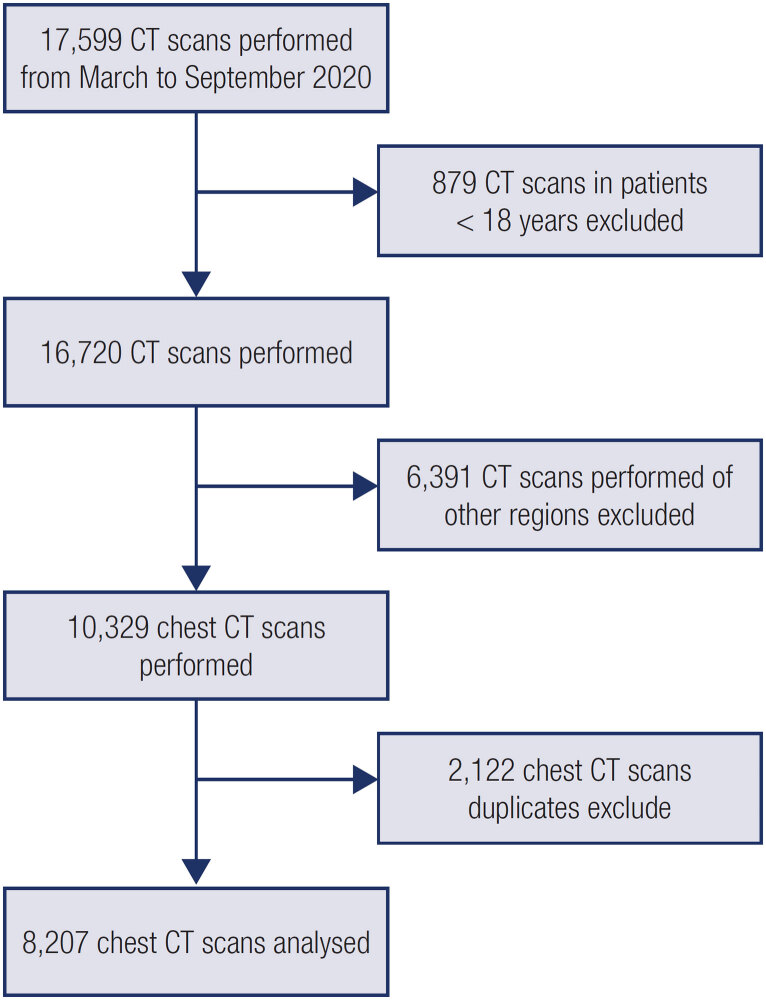
Study flowchart of patient selection.

A total of 10,329 chest CT scans were performed between March and September 2020, and after duplicates were removed, 8,207 exams were included in the study ( Figure 1 ). The median age was 45 years [IQR 35-49 years], 3,025 were under 40 years (36.8%), and 4,667 were female (56.8%). Thirty-eight lesions were found in 36 patients, with an overall prevalence of 0.44% ( Table 1 ). Only two AIs were found in subjects under 40 years ( Table 1 ), and the prevalence increased significantly with age ( Table 2 ). Despite a trend of a higher prevalence in females ( Table 1 ), it was not statistically significant ( Table 2 ). In a logistic regression analysis, a higher detection risk of AI was present in individuals aged 40 and over (RR 9.98; IC 2.39-41.98; p.002), but no relevant risk could be attributed to sex (RR 0.648; IC 0.323-1.299; p.221).

**Table 1:** Demographic and radiological characteristics of the adrenal incidentalomas

Gender		
	Male	12 (33.3%)
	Female	24 (66.7%)
Age group		
	<40 years	2 (5.5%)
	40-60 years	18 (50.0%)
	>60 years	16 (44.4%)
Lateralization		
	Right-side	12 (33.3%)
	Left-side	22 (61.1%)
	Bilateral	2 (5.6%)
Size		
	<1 cm	3 (7.9%)
	1-2 cm	19 (50.0%)
	2-4 cm	11 (28.9%)
	>4 cm	5 (13.1%)
Density		
	<0 HU	11 (29.0%)
	1 to 10 HU	10 (26.3%)
	>10 HU	17 (47.2%)
Calcifications		
	Associated with an adenoma	2 (5.6%)
	Isolated	1 (2.6%)

**Table 2:** Prevalence of adrenal incidentalomas by gender and age group

		Total of patients	Lesions found	P-value
Gender				
	Male	3,540	12 (0.3%)	0.23
	Female	4,667	24 (0.5%)	
Age group				
	<40 years	3,025	2 (0.09%)	
	40 to 60 years	3,220	18 (0.63%)	<0.001
	>60 years	1,962	16 (0.76%)	

**Figure 2:**
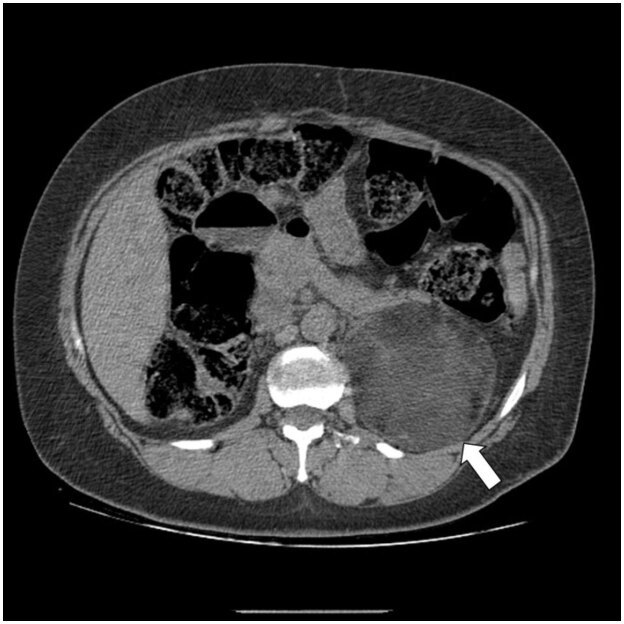
A 52-year-old female patient with a left adrenal lesion of 9.9 cm and -11.9 HU, with features suggesting a myelolipoma (arrow).

**Figure 3:**
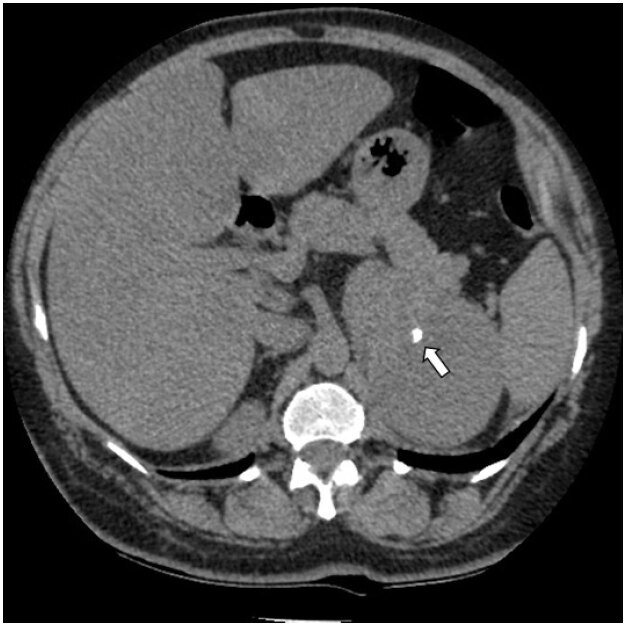
A 61-year-old female patient with a left adrenal lesion of 9.9 cm and 28.6 HU, with a gross calcification inside (arrow).

**Figure 4:**
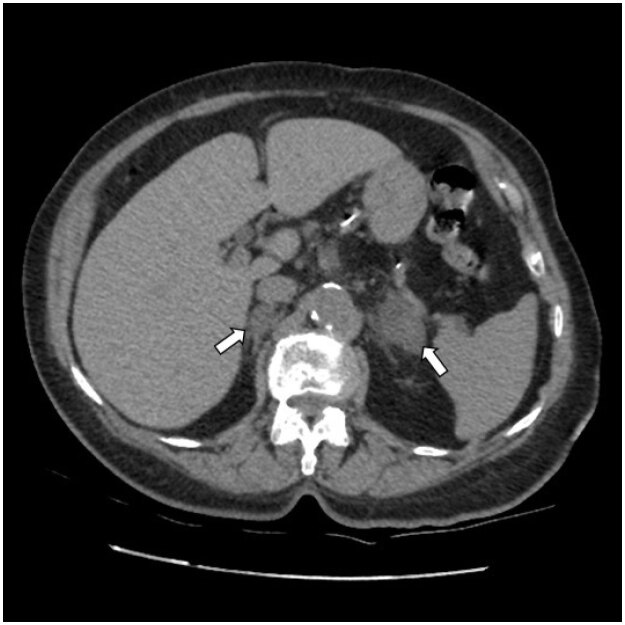
An 84-year-old female patient with bilateral adrenal incidentalomas (arrows). Right-side incidentaloma: 2.1 cm and 3.0 Left- side incidentaloma: 1.26 cm and 8.5 HU.

The mean diameter was 2.53 cm (0.7 to 9.93 cm), and five lesions were above 4 cm ( Table 1 ). Two lesions were 9.9 cm in diameter, both in female subjects, and one of them had radiological features consistent with myelolipoma ( Figure 2 ). Seventeen AIs showed densities above 10 HU ( Table 1 ) in 10 females and 7 males, and only 3 out of 17 had densities greater than 4 cm. Three calcifications were found, and none were associated with pulmonary findings suggestive of tuberculosis or any other pulmonary infection. One of these calcifications was inside a 9.9 cm lesion ( Figure 3 ). Only two patients had bilateral incidentalomas ( Figure 4 ).

## DISCUSSION

The overall prevalence of AI in this study was 0.44%, much less than the mean frequency of 4%-5% previously reported in other studies around the globe (2,3,16). Likewise, a prevalence of 2.5% was found earlier in Brazil (1). Considering that the present study revisits this subject almost two decades later than the latter publication, associated with the availability of high- resolution tomography scans, such a low prevalence was an unexpected finding.

A plausible explanation is that small lesions are frequently missed by the radiologist when reporting an exam completely unrelated to the adrenal gland. Considering the high volume of chest CT scans performed (10,329 exams in 6 months), those professionals were clearly overworked, which may have directed the analysis to the main purpose of the CT scan, i.e., viral pulmonary infection.

Corroborating this argument, Hammarsted and cols. found a frequency of 0.9% of AI in 34,044 abdominal scans in 17 centers during an 18-month interval in Sweden, and after a systematic re-evaluation of 3,801 randomly selected scans, the prevalence increased to 4.5% (16). Likewise, a large “real life” study in Ireland with an unrevised population showed a mean frequency of adrenal findings of 0.81% in the chest and 0.98% in abdominal CT scans, in a total of 3,705 scans performed during a two-year period in a single center (11). It is important to stress the fact that these two studies were not carried out during the pandemic and radiologists were not over a great workload, whereas our study had 8,207 scans performed in a six-month period – three times shorter than the study with the smallest interval. These data emphasize that the prevalence of routinely reported AI in clinical practice is very low and that the impact of AI discovered during the COVID-19 pandemic in the health system should be small.

Regarding the age groups, we found only two lesions (prevalence 0.09%) in subjects below 40 years old, even with a third of the CT scans performed in this age group. The frequency increased significantly from 40 years on, with a risk of an incidental finding nine times greater in this population. This aligns with previous reports, and since a third of our sample (3,025 CT scans) comprised persons younger than 40 years, it is possible to presume that AIs are indeed rare in this age group and that is not a selection bias due to the low frequency of imaging studies performed in this population, as prior publications suggest (4,6,8,11).

Concerning gender, there was a trend toward a higher prevalence in females, which is in agreement with the literature (2,7,17,18). One study in Italy and a previous study in Brazil, however, found a higher frequency in males (1,8). We also had a slight majority of chest CT scans performed on women, which is in disagreement with the expected major severity of COVID-19 in males. A possible explanation is a high number of unnecessary CTs performed in mildly symptomatic patients, and since women are the majority of health care users, it is expected that they would be represented in a larger number in this setting (19). Regarding lateralization, left-side lesions were more frequent, as previously described, and a possible explanation is the better visualization of the left adrenal gland compared to the right, due to organ proximity (1,2,8,16).

A weakness of our study is the lack of clinical information about the persons in which lesions were found. We did not have access to data regarding comorbidities and hormonal investigation triggered by the incidental finding. Another limitation is the retrospective nature of the study and a possible selection bias due to this, which could explain the low rate of AI found. On the other hand, our study confirms that the prevalence of adrenal incidentalomas in routine clinical practice is low, as well as in the age group below 40 years, and the wide usage of imaging exams during the pandemic will not overwhelm the health care system concerning incidental findings.

In conclusion, the overall prevalence of adrenal incidentalomas in routine clinical practice was 0.44%, regarding unselected and unrevised patients in a real- life setting. Incidentalomas in persons below 40 years are extremely rare, with a frequency nine times greater in people aged 40 or more. The widespread use of high-resolution imaging exams during the COVID-19 pandemic should not overload the health care system with a need for specialized follow-up.
